# List-Decoding Capacity of the Gaussian Arbitrarily-Varying Channel †

**DOI:** 10.3390/e21060575

**Published:** 2019-06-07

**Authors:** Fatemeh Hosseinigoki, Oliver Kosut

**Affiliations:** School of Electrical, Computer and Energy Engineering, Arizona State University, Tempe, AZ 85287, USA

**Keywords:** gaussian arbitrarily-varying channel, list-decoding, stochastic encoder, capacity

## Abstract

In this paper, we determine the capacity of the Gaussian arbitrarily-varying channel with a (possibly stochastic) encoder and a deterministic list-decoder under the average probability of error criterion. We assume that both the legitimate and the adversarial signals are restricted by their power constraints. We also assume that there is no path between the adversary and the legitimate user but the adversary knows the legitimate user’s code. We show that for any list size *L*, the capacity is equivalent to the capacity of a point-to-point Gaussian channel with noise variance increased by the adversary power, if the adversary has less power than *L* times the transmitter power; otherwise, the capacity is zero. In the converse proof, we show that if the adversary has enough power, then the decoder can be confounded by the adversarial superposition of several codewords while satisfying its power constraint with positive probability. The achievability proof benefits from a novel variant of the Csiszár-Narayan method for the arbitrarily-varying channel.

## 1. Introduction

An arbitrarily-varying channel (AVC) represents a memoryless channel including unknown parameters that are changing arbitrarily from channel use to channel use. Because these parameters (state) can change arbitrarily, we consider this to be a model for an active adversarial jammer. This adversary sends its signal to restrain the legitimate transmitter and receiver from maintaining reliable communication. In wireless channels, these unpleasant adversaries can easily enter channels, so it is of great importance to study the adversary’s effect on the channel capacity. The capacity of the AVC depends on the coding method (such as randomized coding, stochastic encoder or deterministic coding), the performance criterion (such as average or maximum error probability) and the amount of adversary’s knowledge about the transmitted signal (omniscient, myopic or oblivious adversary). Table 1 provides a summary of various models appearing in the literature, including the one considered here.

Blackwell et al. introduced AVC in Reference [1] and under the average error probability criterion they found the capacity of the discrete memoryless AVC to be given by a min-max expression over the mutual information of input and output. They employed *randomized coding* that is, common randomness between the encoder and the decoder and assumed the jammer to be *oblivious* that is, the jammer does not have any information about the transmitted signal except the code. In Reference [2], it is shown that the min-max expression is equivalent to the corresponding max-min one. Further, in Reference [3], the authors examined that this capacity remains the same even for the maximum error probability criterion, again provided access to common randomness. The case without common randomness between the transmitter and the receiver is referred to as the deterministic code setting. Ahlswede in a notable paper [4] characterized the deterministic capacity of a discrete AVC under the average probability of error through a dichotomy theorem. He proved that the capacity either corresponds to the AVC randomized code capacity or else it equals zero but he did not state any necessary or sufficient condition for which of the two cases prevails. Ericson, in Reference [5], found the necessary condition for the positive alternative by defining *symmetrizability*. A symmetrizable AVC is an AVC in which the adversary can mimic the transmitted signal in order to prevent the decoder from distinguishing between the true message and an adversarial imitation. Thus, he showed that if the deterministic code capacity of an AVC is positive then the channel should be nonsymmetrizable. Later, in Reference [6], a sufficient condition was provided by Csiszár and Narayen stating that if the AVC is nonsymmetrizable then the deterministic code capacity is not zero. Therefore, considering both conditions, the deterministic code capacity of an AVC is positive if and only if the channel is nonsymmetrizable.

The capacity of discrete AVC is also investigated under input and state (or adversarial signal) constraints. Restricted by peak or average input and state cost constraints, the random code capacity of discrete AVC is studied in Reference [7] using the average probability of error as the performance criterion. Furthermore, the second part of Csiszár and Narayan work in Reference [6] focuses on the deterministic code capacity of AVC under input and state constraints for the same performance criterion. They proved that in this case if the capacity is positive then it is less than or equal to the corresponding random code capacity. In particular, with input and state constraints, the capacity can be positive but strictly less than the random code capacity. Note that this does not occur without cost constraints. Csiszár, in Reference [8], extended this result to general input and output alphabets and state sets rather than only finite alphabets and state sets.

There is a wide variety of research on different versions of AVCs under various adversaries model, including References [9,10,11]. Sarwate, in [9], considered a myopic adversary in which there is a discrete memoryless channel (DMC) between the legitimate user and the jammer and the jammer chooses its signal based on this noisy version of the user’s codeword. He found the capacity by minimizing over all DMCs that the jammer can applied by its worst strategies. In Reference [10], single letter characterizations of capacity is obtained in the presence of a delayed adversary which can observe the transmitted signal after a delay. By assuming randomization at the encoder, the capacity is corresponding to the randomized code capacity. B. K. Dey et al., in Reference [11], obtained upper bounds on the capacity of binary channel in the presence of a causal adversary for both maximal and average error probabilities.

On the other hand, AVCs are also studied in network settings throughout [12,13,14,15,16,17,18], such as multiple-access and broadcast channels. The authors in Reference [12,13,14] investigated the capacity of arbitrarily varying multiple-access channels (AVMACs). In Reference [13], it is proved that symmetrizability, which is defined for the two-user AVC, is a sufficient condition for an AVMAC to have empty interior for its deterministic code capacity region. Moreover, throughout [15,16,17,18], the capacity of arbitrarily varying wiretap channels (AVWCs) are determined.

This paper focuses on the Gaussian AVC (GAVC), wherein all alphabets are continuous rather than discrete. Initially, Ahlswede, in Reference [23], studied the capacity of a GAVC in which the adversary chooses the noise variance rather than an additive signal. Hughes and Narayan in Reference [21] determined the randomized code capacity of GAVC under the peak power input and state constraints. They further extended their result in Reference [24] for a vector GAVC. The deterministic code capacity of the GAVC, for the average probability of error, was found in Reference [22]. The authors showed that if the adversary’s power is greater than the legitimate transmitter’s power, then symmetrizability occurs causing the capacity to drop to zero. Note that for a discrete AVC with no cost constraint non-symmetrizability makes the deterministic capacity positive and equal to the randomized capacity [6] (Theorem 1). It is further proved in [6] (Theorem 3) that under input and state cost constraints, non-symmetrizability only results in positive deterministic capacity but it is sometimes strictly less than the randomized capacity. In the Gaussian case, even though there are input and state cost constraints, the behavior is like that of a discrete AVC with no cost constraint, in that if the channel is non-symmetrizable, then its deterministic capacity is positive and equal to the randomized capacity [22].

For the first time, in Reference [19], Hughes showed that using list-decoding, in which the decoder can decode to a small (and bounded) list rather than a unique message estimate, causes positive capacity for most symmetrizable discrete-memoryless AVCs. Intuitively, list-decoding combats the symmetrizing attack by allowing the list to contain the true message as well as the counterfeit(s) generated by the adversary; thus, the receiver can successfully decode even if it cannot specify the correct message. Furthermore, the authors in Reference [20] extended the list-decoding result to the discrete-memoryless AVCs with state constraints. They determined upper and lower bounds on the capacity by introducing two notions of symmetrizability for this channel.

The capacity of AVMAC was also studied with list-decoding in References [25,26]. Sirin Nitinawarat in Reference [25] introduced symmetrizability of an AVMAC and showed that the capacity region for deterministic codes with fixed list-size is empty if the list size is less than the symmetrizability Ω. He obtained that the capacity corresponds to the random code capacity if the list size is greater than ${\left(1+\Omega \right)}^{2}$. H. Boche and R.F. Schaefer in Reference [26] obtained the list capacity region of AVMAC with conferencing encoders which is proved for large list size to be equal to the common randomness assisted capacity region. Moreover, in Reference [27], the authors found the deterministic code and random code capacity regions of arbitrarily varying broadcast channel with receiver side information. By defining a concept of symmetrizability for the channel, they characterized deterministic list codes capacity region as either identical to the random code capacity region or empty.

In this paper, we characterize the capacity of GAVC in Reference [22], using list-decoding for any list size and almost all power values of the transmitter and adversary, a similar result to that of Hughes in Reference [19] which obtained the list capacity for the discrete-memoryless AVC, for which the capacity was determined in Reference [6]. We assume that the encoder may be stochastic—that is, the encoder has access to private randomness—and a deterministic list decoder with constant list size *L*. Under the average probability of error criterion and without common randomness, we obtain the capacity of GAVC with list decoding to be equal to the corresponding randomized code capacity if the list size is greater than the power ratio of the jammer to the legitimate user; otherwise, the capacity is zero. Generally, our problem is a generalized version of the multiple packing of spherical caps problem in Reference [28] with Gaussian noise; although, they assumed maximal probability of error as the performance criterion. Their upper bound, which is only calculated for the noiseless case, depends on the list size *L* even in the asymptotic case. However, we only have list size in our symmetrizability conditions rather than the capacity itself.

In our converse proof (in Section 4), the adversary focuses on two possible strategies, one of which is simply sending Gaussian noise which causes the channel to act as a standard Gaussian channel with increased noise variance. The second strategy for the adversary is to transmit the superposition of some random (counterfeit) codewords, which is shown to be possible with positive probability if its power is large enough. In our achievability proof (in Section 5), we employ Gaussian codewords with a particular version of minimum distance list-decoding based on typicality. We extend the scheme of Reference [22] to show that with high probability a random Gaussian codebook has desirable properties to make the probability of error zero. However, our achievability proof originates from the idea of Reference Reference [6] based on typical sets, rather than the geometric approach of Reference [22]. This scheme allows us for simpler characterizations of codebook constraints. It is worth mentioning that we prove the achievability for the deterministic encoder since it suffices to achieve a rate even by a deterministic code, that is any deterministic code is a realistic value of a stochastic code. Our converse and achievability proofs apply for any list size; our previous work [29] provided proofs only for list size L=2.

*Notations:* Upper case letters denote random variables while lower case letters specify a deterministic value or the realization of a random variable. Bold letters denotes *n*-length vectors. We indicate inner product and 2-norm by 〈·,·〉 and ∥·∥, respectively. We use ${\left|\cdot \right|}^{+}$ and E[·] to denote the positive-part function and the expectation, respectively. Also, for an integer *N*, notation [N] represents the set {1,2,3,…,N}. Notation $I_{n}$ and $1_{n}$ stand for the identity matrix of size *n* and a vector of size *n* with *n* ones elements, respectively. For a vector v, we use superscript $v^{T}$ to denote its transpose. Both log(·) and exp(·) functions has base 2, so we define the Gaussian channel capacity function $C\left(x\right)=\frac{1}{2}\log\left(1+x\right)$.

## 2. Problem Statement

A GAVC is a modified standard point-to-point Gaussian channel in the presence of an additive arbitrarily chosen adversary signal. Both transmitter and receiver do not know anything about the adversary signal and the adversary do not have any information about the transmitted signal except the codebooks. The received signal is given by

(1) $$\begin{array}{c} \begin{array}{c} Y=x+s+V \end{array} \end{array}$$

where the *n*-length vector x is the legitimate transmitter’s signal, s represents the independent adversary signal and noise V is a sequence of *n*-length i.i.d. zero mean Gaussian random variables with variance $\sigma^{2}$, independent of x and s.

We have the assumption of peak power constraints for the transmitter and adversary signals respectively as ${∥x∥}^{2}\leq \;nP$ and ${∥s∥}^{2}\leq n\Lambda$. In addition, the transmitter and receiver are assumed to know the three parameters *P*, Λ and $\sigma^{2}$.

An $\left(n,N,N_{r},L\right)$ stochastic list code for the GAVC is given by:

- Message set M=[N] and encoder private randomness set $K=[N_{r}]$,
- Stochastic encoding function $x\left(M,K\right):M\times K\to R^{n}$,
- List decoding function $\phi \left(y\right):R^{n}\to D_{L}=\left\{L\;:\;L\;\subset \;\left[N\right],|L|\;\leq \;L\right\}$,

where the rate of the code is $R=\frac{1}{n}\log\left(N/L\right)$. The transmitter encodes the message *M* and its private randomness *K* to x(M,K) where *M* and *K* are chosen uniformly respectively from sets M and K. At the receiver, signal Y is decoded by a deterministic function ϕ to the set $D_{L}$ which is the set of all subsets of [N] with cardinality at most *L*. In other words, *L* is the size of the list decoder.

First, define the probability of error e(s,i) for a specific message i∈[N] in the presence of a specific adversary signal $s\in R^{n}$ as the probability that i∉ϕ(y). Therefore, the average probability of error for s is given by

(2) $$\begin{array}{c} \bar{e}\left(s\right)=\frac{1}{N}\sum_{i=1}^{N}e\left(s,i\right). \end{array}$$

Finally, the overall probability of error $P_{e}^{\left(n\right)}$ is obtained by maximizing over all possible choices of jammers’ sequences s satisfying peak power constraint ${∥s∥}^{2}\leq n\Lambda$. Suppose *r* is rate of private randomness. Given *L* and *r*, rate *R* is *achievable* if there exists a sequence of $\left(n,L2^{nR},2^{nr},L\right)$ codes such that $\lim_{n\to \infty }P_{e}^{\left(n\right)}=0$. The list-code capacity C(L,r) is the supremum of all achievable rates given *L* and *r*.

## 3. Main Results

**Theorem** **1.** *The list-code capacity of GAVC is given by*

(3) $$\begin{array}{c} C\left(L,r\right)=\left\{\begin{array}{cc} C\left(\frac{P}{\Lambda +\sigma^{2}}\right), & L>\frac{\Lambda }{P}\\ 0, & L<\frac{\Lambda }{P}. \end{array}\right. \end{array}$$

*Note that the capacity for* Λ=LP *is unsolved.*

**Remark** **1.** *Note that this result holds for all r, including* r=0 *which corresponds to a deterministic encoder. That is, the capacity does not depend on the amount of private randomness.*

**Remark** **2.** *The condition on the ratio*$\frac{\Lambda }{P}$*determines whether it is possible for the adversary to launch a symmetrizing attack, wherein it transmits a superposition of codewords. Since each codeword has power P, the most codewords that the adversary can superpose while obeying its power constraint of* Λ *is the* $\left\lfloor \frac{\Lambda }{P}\right\rfloor$*. Thus, if the allowable list size is greater than* $\frac{\Lambda }{P}$*, then even under this attack the decoder can output a list made up of the true message and the superposed codewords selected by the adversary. Of course, the decoder does not know which is which but it can still guarantee that the true message is in the list. Thus, the worst the adversary can do is to act as an extra additive Gaussian noise with variance* Λ*, so the capacity is equal to the capacity of a standard Gaussian channel with increased noise variance as in* $C(\frac{P}{\Lambda +\sigma^{2}})$*. However, if the allowable list size is less than* $\frac{\Lambda }{P}$*, then there are too many possibilities for the decoder to decode correctly, so the capacity is zero. Note that none of this depends on the channel noise, so* $\sigma^{2}$ *does not come into play in the condition on L.*

**Remark** **3.** *For the achievability proof, we make no assumptions about what the adversary does. However, for the converse proof, it is allowable to weaken the adversary by making certain assumptions about its behavior, because doing so can only increase the achievable rates. Since the converse is an upper bound on achievable rates, weakening the adversary in this manner still yields a valid upper bound.*

In our proofs in Section 4 and Section 5, without loss of generality we restrict ourselves to the transmitter’s power P=1 which can be done by scaling the output signal.

## 4. Converse Proof

Without loss of generality, suppose P=1. For the first case where Λ<L, we assume that we have a code $\left(n,L2^{nR},2^{nr},L\right)$ with vanishing probability of error. Since these codes must function for arbitrary jamming signals, we may derive an outer bound by assuming the adversary transmits Gaussian noise with variance Λ−γ for any γ>0 or 0 if Gaussian realization has power greater than Λ. By the law of large numbers, with high probability the resulting channel is equivalent to a standard Gaussian channel with noise variance $\sigma^{2}+\Lambda -\gamma$. Thus, since γ can be chosen arbitrarily small, from the capacity of a non-adversarial Gaussian channel,

(4) $$C\left(L,r\right)\leq C\left(\frac{1}{\sigma^{2}+\Lambda }\right).$$

Now, assume the symmetrizable case where Λ>L. In order to show C(L,r)=0, first consider a sequence of stochastic codebooks and probability of error $P_{e}^{\left(n\right)}$. We claim that if R>0 and the jammer has the following strategy, then $P_{e}^{\left(n\right)}$ is bounded away from zero for sufficiently large *n*: The jammer randomly and uniformly chooses *L* messages $M_{1},\ldots ,M_{L}$ from $\left[L2^{nR}\right]$ and also *L* private keys $K_{1},\ldots ,K_{L}$ from $\left[2^{nr}\right]$ where $M_{i}$ and $K_{i}$ are independent. Note that the jammer knows the transmitted codebook. The jammer then constructs

(5) $$Z=x\left(M_{1},K_{1}\right)+\ldots +x\left(M_{L},K_{L}\right)-L\mu$$

where $\mu \in R^{n}$ is a constant vector that we will choose later. The jammer transmits S=Z if ${∥Z∥}^{2}\leq n\Lambda$ or else transmits S=0. In the former case, the received signal is

(6) $$\begin{array}{cc} Y & =x\left(M_{0},K_{0}\right)+x\left(M_{1},K_{1}\right)+\ldots +x\left(M_{L},K_{L}\right)-L\mu +V \end{array}$$

(7) $$\begin{array}{c} =\mu +\sum_{i=0}^{L}\left(x\left(M_{i},K_{i}\right)-\mu \right)+V \end{array}$$

where $M_{0}$ is the true message. If $M_{0},M_{1},\ldots ,M_{L}$ are all different and for all sets D⊂{0,1,…,L} with |D|=L,

(8) $${∥\sum_{i\in D}x\left(M_{i},K_{i}\right)-L\mu ∥}^{2}\leq n\Lambda ,$$

then from the decoder’s perspective, any *L* of the L+1 messages might have been forged by the adversary. Therefore, the list decoder with list size at most *L* has a probability of error at least $\frac{1}{L+1}$; that is, the probability that the decoder chooses *L* from the L+1 messages that does not contain the true message $M_{0}$. That is,

(9) $$\begin{array}{l} P_{e}^{\left(n\right)}\geq \frac{1}{L+1}P({∥\sum_{i\in D}x\left(M_{i},K_{i}\right)-L\mu ∥}^{2}\;\leq \;n\Lambda \\ \;\;\mathrm{for}\;\mathrm{all}\;D\subset \left\{0,1,\ldots ,L\right\}\;\mathrm{with}\;|D|=L,\;\mathrm{and}\;M_{0},M_{1},\ldots ,M_{L}\;\mathrm{are}\;\mathrm{distinct}) \end{array}$$

(10) $$\begin{array}{l} \geq \frac{1}{L+1}[P\left({∥\sum_{i\in D}X_{i}-\;L\mu ∥}^{2}\leq n\Lambda \;\mathrm{for}\;\mathrm{all}\;D\subset \left\{0,1,\ldots ,L\right\}\;\mathrm{with}\;\left|D\right|=L\right)\;\\ \;-\left(\;1\;-\;\frac{L2^{nR}-1}{L2^{nR}}\cdot \frac{L2^{nR}-2}{L2^{nR}}\cdots \;\frac{L2^{nR}-L}{L2^{nR}}\right)] \end{array}$$

where $X_{i}=x\left(M_{i},K_{i}\right)$ and the second term in (10) shows the probability of messages $M_{0},\ldots ,M_{L}$ not being distinct which tends to zero as n→∞. Note that $X_{0},X_{1},\ldots ,X_{L}$ are independent and each distributed as a transmitted sequence from the code. We proceed to show that there exists a choice of μ based only on the codebook such that (10) is bounded away from zero for sufficiently large *n* if R>0.

Let

(11) $$\alpha^{⋆}\;=\;\inf\left\{\;\alpha \;:\;\underset{n\to \infty }{lim inf}\;\max_{\mu \in R^{n}}P(∥X_{0}-\mu {∥}^{2}\leq n\alpha )>0\right\}.$$

Note that $\alpha^{⋆}\leq 1$, since by the power constraint we always have $P(∥X_{0}{∥}^{2}\leq n)=1$. Fix any δ>0 and let $\alpha =\alpha^{⋆}+\delta /2$. Let

(12) $$\gamma =\underset{n\to \infty }{lim inf}\;\max_{\mu \in R^{n}}P(∥X_{0}-\mu {∥}^{2}\leq n\alpha ).$$

Since $\alpha >\alpha^{⋆}$ we have γ>0. Thus for *n* sufficiently large, there exists $\mu \in R^{n}$ such that

(13) $$P(∥X_{0}-\mu {∥}^{2}\leq n\alpha )\geq \gamma -\delta .$$

This μ is the one to be used by the jammer in (5). Let $B_{n}$ be the set of all x that satisfy ${∥x-\mu ∥}^{2}\leq n\alpha$. Note that $P(X_{0}\in B_{n})\geq \gamma -\delta$.

Since $\alpha -\delta <\alpha^{⋆}$, by the definition of $\alpha^{⋆}$,

(14) $$\underset{n\to \infty }{lim inf}\;\max_{\mu^{\prime }\in R}\;P(∥X_{0}-\mu^{\prime }{∥}^{2}\leq n\left(\alpha -\delta \right))=0.$$

Specifically, there exists *n* sufficiently large so that for all $\mu^{\prime }\in R^{n}$,

(15) $$P(∥X_{0}-\mu^{\prime }{∥}^{2}\leq n\left(\alpha -\delta \right))\leq \delta .$$

Fix any $x_{1}\in B_{n}$ and consider those $x\in B_{n}$ such that

(16) $$\langle x-\mu ,x_{1}-\mu \rangle >n\sqrt{\delta \alpha }$$

which implies

(17) $$\begin{array}{cc} {∥\mu +\sqrt{\delta \alpha^{-1}}\left(x_{1}-\mu \right)-x∥}^{2} & ={\;∥x\;-\;\mu ∥}^{2}\;+\;\delta \alpha^{-1}{∥x_{1}\;-\;\mu ∥}^{2}\;-\;2\sqrt{\delta \alpha^{-1}}\langle x\;-\;\mu ,x_{1}\;-\;\mu \rangle \end{array}$$

(18) $$\begin{array}{c} <n\alpha +n\delta -n2\delta \end{array}$$

(19) $$\begin{array}{c} =n(\alpha -\delta ). \end{array}$$

Thus, we obtain the following by applying (15) with $\mu^{\prime }=\mu +\sqrt{\delta \alpha^{-1}}\left(x_{1}-\mu \right)$ as

(20) $$\begin{array}{c} P\left(\langle X_{0}-\mu ,X_{1}-\mu \rangle >n\sqrt{\delta \alpha },\;X_{0},X_{1}\in B_{n}\right)\leq \max_{x_{1}\in B_{n}}\;P\left(\langle X_{0}-\mu ,x_{1}-\mu \rangle >n\sqrt{\delta \alpha },\;X_{0}\in B_{n}\right)\leq \delta . \end{array}$$

Moreover, if $x_{1},\ldots ,x_{L}\;\in \;B_{n}$ satisfy $\langle x_{i}-\mu ,x_{j}-\mu \rangle \;\leq \;n\sqrt{\delta \alpha }$ for all i≠j∈{1,…,L}, then

(21) $$\begin{array}{cc} ∥x_{1}+\ldots +x_{L}{-L\mu ∥}^{2} & =\sum_{i=0}^{L-1}{∥x_{i}-\mu ∥}^{2}+2\sum_{i=0}^{L-2}\sum_{j=i+1}^{L-1}\langle x_{i}-\mu ,x_{j}-\mu \rangle \end{array}$$

(22) $$\begin{array}{c} \leq n\left(L\alpha +L\left(L-1\right)\sqrt{\delta \alpha }\right) \end{array}$$

(23) $$\begin{array}{c} \leq n\left(L+L\delta +L\left(L-1\right)\sqrt{\delta \alpha }\right) \end{array}$$

(24) $$\begin{array}{c} <n\Lambda \end{array}$$

where (23) holds since $\alpha <\alpha^{⋆}+\delta \leq 1+\delta$ and (24) holds for sufficiently small δ and by assumption Λ>L. Now we have

(25) $$\begin{array}{c} P\left({∥\sum_{i\in D}X_{i}-\;L\mu ∥}^{2}\leq n\Lambda \;\mathrm{for}\;\mathrm{all}\;\mathrm{distinct}\;\mathrm{set}\;D\subset \left\{0,1,\ldots ,L\right\}\;\mathrm{with}\;\left|D\right|=L\right)\\ \;\geq P\left(\langle X_{i}-\mu ,X_{j}-\mu \rangle \leq n\sqrt{\delta \alpha }\;\mathrm{for}\;\mathrm{all}\;i,j\in \left\{0,1,\ldots ,L\right\},i\neq j,X_{0},X_{1},\ldots ,X_{L}\in B_{n}\right) \end{array}$$

(26) $$\begin{array}{c} \;\geq P{\left(X_{0}\in B_{n}\right)}^{\left(L+1\right)}-\frac{L(L+1)}{2}\;P\left(\langle X_{0}-\mu ,X_{1}-\mu \rangle >n\sqrt{\delta \alpha },X_{0},X_{1}\in B_{n}\right) \end{array}$$

(27) $$\begin{array}{c} \;\geq {\left(\gamma -\delta \right)}^{\left(L+1\right)}-\frac{L(L+1)\delta }{2} \end{array}$$

where (25) follows from the analysis leading to (24), (26) follows from the union bound and the fact that $X_{0},X_{1},\ldots ,X_{L}$ are independent and (27) follows from the lower bound on the probability of $B_{n}$ and (20). For sufficiently small δ, (27) is bounded away from zero, so by (10), $P_{e}^{\left(n\right)}$ is also bounded away from zero for sufficiently large *n* if R>0.

## 5. Achievability Proof

Before proceeding to the proof, let define the typical set for Gaussian random variables $X_{1},\ldots ,X_{k}$ as:

(28) $$\begin{array}{c} T_{\epsilon }^{\left(n\right)}\left(X_{1},\ldots ,X_{k}\right)\;=\;\left\{\;\left(x_{1},\ldots ,x_{k}\right)\;:\;E\left(X_{i}X_{j}\right)-\epsilon \;\leq \;\frac{1}{n}\langle x_{i},x_{j}\rangle \;\leq \;E\left(X_{i}X_{j}\right)+\epsilon \;\mathrm{for}\;\mathrm{all}\;i,j\;\in \;\left[1\;:\;k\right]\;\right\}. \end{array}$$

Without loss of generality, assume P=1, r=0 and

(29) $$\begin{array}{c} \Lambda <L, \end{array}$$

(30) $$\begin{array}{c} R<C\left(\frac{1}{\Lambda +\sigma^{2}}\right). \end{array}$$

Note that assuming r=0 makes the code deterministic. Thus, it suffices to achieve the list-code capacity by a $\left(n,L2^{nR},L\right)$ deterministic code construction as follows:

*Codebook generation:* Fix $\epsilon >\epsilon^{\prime }>\gamma >0$. We generate $L2^{nR}$ i.i.d zero mean Gaussian sequences X(m) with variance (1−γ) for each $m\in [L2^{nR}]$.

*Encoding:* The transmitter sends X=X(m) if its power is less than 1, otherwise it sends zero.

*Decoding:* First, given y, for 1≤ℓ≤L let set $S_{\ell }$ be the set of *ℓ*-tuple messages $\left(m_{1},\ldots ,m_{\ell }\right)$ that $\left(x\left(m_{1}\right),\ldots ,x\left(m_{\ell }\right),y\right)\in T_{\epsilon }^{\left(n\right)}\left(X_{1},\ldots ,X_{\ell },Y\right)$ for any set of zero-mean Gaussian variables $\left(X_{1},\ldots ,X_{\ell },Y\right)$ such that

(31) $$\mathrm{Cov}\left(X_{1},\ldots ,X_{\ell },Y\right)={\left[\begin{array}{cc} I_{\ell } & 1_{\ell }^{T}\\ 1_{\ell } & A \end{array}\right]}_{\left(\ell +1)\times (\ell +1\right)}$$

and $1\leq A\leq 1+\sigma^{2}+\Lambda$.

Now we define the decoding function as

(32) $$\begin{array}{c} \phi \left(y\right)=\underset{L\in {⋃}_{\ell =1}^{L}S_{\ell }}{arg min}∥y-\sum_{m\in L}x\left(m\right)∥. \end{array}$$

where we choose between multiple minimizing L arbitrarily.

*Analysis of the probability of error:* To prove that the constructed code is achievable meaning that the probability of error tends to zero as n→∞, we utilize several lemmas including the following. We provide some necessary codebook properties that hold with high probability in Lemma 1, the proof for which is in the Appendix A.

**Lemma** **1.** *Let* X(m) *for* m∈[N] *,* $N=L2^{nR}$ *, be the Gaussian codebook described above. With probability approaching 1 as* n→∞ *, the codebook satisfies the following, for any* x,s *where* ${∥s∥}^{2}\leq n\Lambda$ *and any zero-mean jointly Gaussian random vector* $\left(X,X_{1},\ldots ,X_{\ell },S\right)$ *with positive definite covariance matrices with diagonals at most* (1,1,…,1,Λ) *for all* 1≤ℓ≤L *:*

(33) $$\begin{array}{c} \frac{1}{N}\left|\left\{\;m\;:\;\left(x\left(m\right),s\right)\notin \;\underset{\begin{array}{c} (X,S)\;\mathrm{independent}:\\ EX^{2}=1,ES^{2}\leq \Lambda \end{array}}{⋃}\;T_{\epsilon^{\prime }}^{\left(n\right)}\left(X,S\right)\right\}\right|\;\leq \;\exp\left(-n\delta \left(\epsilon \right)\right), \end{array}$$

(34) $$\begin{array}{c} |\left\{m_{1}:\left(x,x\left(m_{1}\right),s\right)\in T_{\epsilon }^{\left(n\right)}\left(X,X_{1},S\right)\right\}|\leq \exp\left\{n\left[|R-I\left(X_{1};XS\right){|}^{+}+\delta \left(\epsilon \right)\right]\right\}, \end{array}$$

(35) $$\begin{array}{c} \frac{1}{N}|\left\{\;m\;:\;\left(x\left(m\right),x\left(m_{1}\right),s\right)\;\in \;T_{\epsilon }^{\left(n\right)}\;\left(X,X_{1}\;,S\right)\;\mathrm{for}\;\mathrm{some}\;m_{1}\;\neq \;m\;\right\}|\leq \;2\exp\left\{\;-n\delta \left(\epsilon \right)/2\;\right\},\\ \;if\;I\left(X;X_{1}S\right)\;\geq \;{\left|R\;-\;I\left(X_{1};S\right)\right|}^{+}\;\;+\;\delta \left(\epsilon \right), \end{array}$$

(36) $$\begin{array}{c} \left|\left\{\;\left(m_{1},\ldots ,m_{\ell }\right)\;:\;\left(x,x\left(m_{1}\right),\ldots ,x\left(m_{\ell }\right),s\right)\in T_{\epsilon }^{\left(n\right)}\left(X,X_{1},\ldots ,X_{\ell },S\right)\right\}\right|\leq \exp\left\{n\delta (\epsilon )\right\}\\ \;if\;R<\min_{k\in \{1,\ldots ,\ell \}}I\left(X_{k};S\right), \end{array}$$

(37) $$\begin{array}{c} \frac{1}{N}|\left\{m:\left(x\left(m\right),x\left(m_{1}\right),\ldots ,x\left(m_{\ell }\right),s\right)\in T_{\epsilon }^{\left(n\right)}\left(X,X_{1},\ldots ,X_{\ell },S\right)\;for\;some\;m_{1},\ldots ,m_{\ell }\neq m\right\}|\\ \;\leq \exp\left\{-n\delta \left(\epsilon \right)/2\right\},\;if\;I\left(X;X_{1}\ldots X_{\ell }S\right)\;\geq \;\delta \left(\epsilon \right)\;and\;R\;<\;\min_{k\in \{1,\ldots ,\ell \}}I\left(X_{k};S\right). \end{array}$$

The following lemmas can be easily generalized for Gaussian random variables by following the corresponding lemmas in Reference [30] for discrete memoryless random variables.

*Conditional Typicality Lemma*: Let (X,Y)∼f(x,y) be jointly Gaussian random variables. Suppose that $x\in T_{\epsilon }^{\left(n\right)}\left(X\right)$ and $Y∼f\left(y|x\right)=\prod_{i=1}^{n}f_{Y|X}\left(y_{i}|x_{i}\right)$. Then, for every $\epsilon >\epsilon^{\prime }$,

(38) $$\begin{array}{c} \lim_{n\to \infty }P\left\{\left(x,Y\right)\in T_{\epsilon }^{\left(n\right)}\left(X,Y\right)\right\}=1. \end{array}$$

*Joint Typicality Lemma*: Let (X,Y,Z)∼f(x,y,z) be jointly Gaussian random variables. If $\left(\tilde{x},\tilde{y}\right)$ is a pair of arbitrary sequences and $\tilde{Z}∼\prod_{i=1}^{n}f_{Z|X}\left({\tilde{z}}_{i}|{\tilde{x}}_{i}\right)$ then there exists δ(ϵ)>0 that tends to zero as ϵ→0 such that

(39) $$\begin{array}{c} P\left\{\left(\tilde{x},\tilde{y},\tilde{Z}\right)\;\in \;T_{\epsilon }^{\left(n\right)}\left(X,Y,Z\right)\right\}\leq \exp\left(-n\left(I\left(Y;Z|X\right)\;-\;\delta \left(\epsilon \right)\right)\right). \end{array}$$

Assume that the legitimate user transmits message *M*. Then, the probability of error is upper bounded by the sum of the following *L* error events probabilities:

(40) $$\begin{array}{l} P_{\ell }=\;P\{\;∥Y\;-\;x\left(m_{1}\right)\;-\ldots -\;x\left(m_{\ell }\right){∥}^{2}\;\leq \;\min_{\begin{array}{c} {\hat{m}}_{1},\ldots ,{\hat{m}}_{\ell }:\\ \left(M,{\hat{m}}_{1},\ldots ,{\hat{m}}_{\ell }\right)\in S_{\ell +1} \end{array}}∥Y\;\;-\;x\left(M\right)\;-\;x\left({\hat{m}}_{1}\right)\;-\ldots -\;x\left({\hat{m}}_{\ell }\right){∥}^{2}\\ \;\;\mathrm{for}\;\mathrm{some}\;\left(m_{1},\ldots ,m_{\ell }\right)\in S_{\ell },m_{i}\neq M\;\mathrm{for}\;\mathrm{all}\;i\in \left[\ell \right]\}\;\mathrm{for}\;1\leq \ell <L, \end{array}$$

(41) $$\begin{array}{cc} P_{L} & =\;P\left\{\exists \left(m_{1},\ldots ,m_{L}\right)\in S_{L}:m_{\ell }\neq M,\;\mathrm{for}\;\mathrm{all}\;\ell \in \left[L\right]\right\}. \end{array}$$

By Lemma 1, we may assume we have a deterministic codebook that satisfies (33)–(37). Consider any state sequence s. By (33), with high probability $\left(x\left(M\right),s\right)\in T_{\epsilon^{\prime }}^{\left(n\right)}\left(X,S\right)$ where (X,S) are independent and $EX^{2}=1,ES^{2}\leq \Lambda$ (33). Thus, by the conditional typicality lemma, for every $\epsilon >\epsilon^{\prime }$ with high probability $\left(x_{i},s,V\right)\in T_{\epsilon }^{\left(n\right)}\left(X,S,V\right)$ where (X,S,V) are mutually independent and $EV^{2}=\sigma^{2}$.

We first bound probability event $P_{\ell }$ for 1≤ℓ<L. Define the shorthand ${\vec{X}}_{\ell }=\left(XX_{1}\ldots X_{\ell }SV\right)$. Let $V_{\ell }$ denote a finite set of Gaussian distributions of ${\vec{X}}_{\ell }$ that is ϵ-dense in the set of all Gaussian distributions of ${\vec{X}}_{\ell }$ with variances at most $\left(1,1,\ldots ,1,\Lambda ,\sigma^{2}\right)$. Note that the cardinality of $V_{\ell }$ does not depend on *n*. We may upper bound $P_{\ell }$ by

(42) $$\sum_{{\vec{X}}_{\ell }\in V_{\ell }}\frac{1}{N}\sum_{m=1}^{N}e_{{\vec{X}}_{\ell }}\left(m,s\right)$$

where

(43) $$\begin{array}{c} e_{{\vec{X}}_{\ell }}\left(m,s\right)=P\{\left(x\left(m\right),x\left(m_{1}\right),\ldots ,x\left(m_{\ell }\right),s,V\right)\in T_{\epsilon }^{\left(n\right)}\left({\vec{X}}_{\ell }\right),\\ ∥x\left(m\right)+s\;+\;V\;-\;x\left(m_{1}\right)-\ldots -\;x\left(m_{\ell }\right){∥}^{2}\leq \min_{\begin{array}{c} {\hat{m}}_{1},\ldots ,{\hat{m}}_{\ell }:\\ \left(m,{\hat{m}}_{1},\ldots ,{\hat{m}}_{\ell }\right)\in S_{\ell +1} \end{array}}{∥s+V-x\left({\hat{m}}_{1}\right)-\ldots -x\left({\hat{m}}_{\ell }\right)∥}^{2}\\ \;\mathrm{for}\;\mathrm{some}\;\left(m_{1},\ldots ,m_{\ell }\right)\in S_{\ell }\;\mathrm{and}\;m_{i}\neq m\;\mathrm{for}\;\mathrm{all}\;i\in \left[\ell \right]\}. \end{array}$$

We will show that $\frac{1}{N}\sum_{m=1}^{N}e_{{\vec{X}}_{\ell }}\left(m,s\right)\to 0$ for all Gaussian vectors ${\vec{X}}_{\ell }$ (whether or not they are in $V_{\ell }$). We may restrict ourselves to ${\vec{X}}_{\ell }$ where

(44) $$\begin{array}{c} (X,S,V)\;\mathrm{are}\;\mathrm{mutually}\;\mathrm{independent}, \end{array}$$

(45) $$\begin{array}{c} EX^{2}=EX_{1}^{2}=\ldots =EX_{\ell }^{2}=1,\;EV^{2}=\sigma^{2},\;ES^{2}\leq \Lambda \end{array}$$

(46) $$\begin{array}{c} \left(X_{i},X+S+V-X_{i}\right)\;\mathrm{are}\;\mathrm{independent}\;\mathrm{for}\;\mathrm{all}\;i\in \left[\ell \right], \end{array}$$

(47) $$\begin{array}{c} E{\left(X+S+V-X_{i}\right)}^{2}\leq \Lambda +\sigma^{2}\;\mathrm{for}\;\mathrm{all}\;i\in \left[\ell \right], \end{array}$$

where (44) holds since the legitimate transmitter, the jammer and the noise are independently generated, (45) follows from the assumptions for ${\vec{X}}_{\ell }$, (46) corresponds to $EX_{i}\left(Y-X_{i}\right)=0$ following from (31) and the assumption that $\left(m_{1},\ldots ,m_{\ell }\right)\in S_{\ell }$ and (47) is obtained by (31) as follows:

(48) $$\begin{array}{cc} E{\left(X+S+V-X_{i}\right)}^{2} & =E{\left(Y-X_{i}\right)}^{2} \end{array}$$

(49) $$\begin{array}{c} =EY^{2}+EX_{i}^{2}-2EYX_{i} \end{array}$$

(50) $$\begin{array}{c} \leq 1+\sigma^{2}+\Lambda +1-2 \end{array}$$

(51) $$\begin{array}{c} =\Lambda +\sigma^{2}. \end{array}$$

Now, suppose

(52) $$I(XV;SX_{1}\ldots X_{\ell })=0.$$

Then we would have $EXX_{i}=0$ for all i∈[ℓ]. Thus, $\left(X,X_{1},\ldots ,X_{\ell },S+V-X_{1}-\ldots -X_{\ell }\right)$ are mutually independent since

(53) $$\begin{array}{c} EX\left(S+V-X_{1}-\ldots -X_{\ell }\right)=EX\left(S+V\right)=0, \end{array}$$

and

(54) $$\begin{array}{cc} EX_{i}\left(S+V-X_{1}-\ldots -X_{\ell }\right) & =EX_{i}\left(Y-X-X_{1}-\ldots -X_{\ell }\right) \end{array}$$

(55) $$\begin{array}{c} =EX_{i}\left(Y-X_{i}\right) \end{array}$$

(56) $$\begin{array}{c} =0,\;\mathrm{for}\;\mathrm{all}\;i\in [\ell ], \end{array}$$

where (53) follows from (44), (55) and (56) follow from (31) and $EXX_{i}=0$. Hence, if the message $\left(m_{1},\ldots ,m_{\ell }\right)$ satisfies the conditions in the probability in (43), then $\left(m,m_{1},\ldots ,m_{\ell }\right)\in S_{\ell +1}$. This implies that $\left({\hat{m}}_{1},\ldots ,{\hat{m}}_{\ell }\right)$ takes on the value $\left(m_{1},\ldots ,m_{\ell }\right)$ in the minimum, so $∥Y-x\left(m_{1}\right)-\ldots -x\left(m_{\ell }\right){∥}^{2}\leq {∥Y-x\left(m_{1}\right)-\ldots -x\left(m_{\ell }\right)-x\left(M\right)∥}^{2}$ and so we must have

(57) $$E{\left(X+S+V-X_{1}-\ldots -X_{\ell }\right)}^{2}\leq E{\left(S+V-X_{1}-\ldots -X_{\ell }\right)}^{2}.$$

However, this contradicts the assumptions that *X* is independent from $S,X_{1},\ldots ,X_{\ell },V$, since

(58) $$\begin{array}{cc} E{\left(X+S+V-X_{1}-\ldots ,X_{\ell }\right)}^{2} & =EX^{2}+E{\left(S+V-X_{1}-\ldots ,X_{\ell }\right)}^{2} \end{array}$$

(59) $$\begin{array}{c} =1+E{\left(S+V-X_{1}-\ldots ,X_{\ell }\right)}^{2}. \end{array}$$

Therefore, the assumption in (52) is false and there exists η>0 such that

(60) $$\eta \leq I\left(XV;SX_{1}\ldots X_{\ell }\right)=I\left(XV;X_{1}\ldots X_{\ell }|S\right).$$

Now, consider the following two cases.

Case (a): $R<\min\{I\left(X_{1};S\right),\ldots ,I\left(X_{\ell };S\right)\}$. By (37), we only need to consider distributions where

(61) $$I\left(X;X_{1}\ldots X_{\ell }S\right)<\delta \left(\epsilon \right).$$

Then for any m,s

(62) $$\begin{array}{cc} e_{{\vec{X}}_{\ell }}\left(m,s\right) & \leq \sum_{\begin{array}{c} m_{1},\ldots ,m_{\ell } \end{array}}P\left\{\;\left(x\left(m\right),x\left(m_{1}\right),\ldots ,x\left(m_{\ell }\right),s,V\right)\;\in \;T_{\epsilon }^{\left(n\right)}\left(X,X_{1},\ldots ,X_{\ell },S,V\right)\;\right\} \end{array}$$

(63) $$\begin{array}{c} \leq \exp\left\{-n\left(I\left(V;X_{1}\ldots X_{\ell }|XS\right)-\delta \left(\epsilon \right)\right)\right\} \end{array}$$

(64) $$\begin{array}{c} =\exp\{-n\left(I\left(XV;X_{1}\ldots X_{\ell }|S\right)-I\left(X;X_{1}\ldots X_{\ell }|S\right)-\delta \left(\epsilon \right)\right)\} \end{array}$$

(65) $$\begin{array}{c} \leq \exp\{-n(\eta -2\delta (\epsilon ))\} \end{array}$$

where in (62) the sum is over all $m_{1},\ldots ,m_{\ell }:\left(x\left(m\right),x\left(m_{1}\right),\ldots ,x\left(m_{\ell }\right),s\right)\in T_{\epsilon }^{\left(n\right)}\left(X,X_{1},\ldots ,X_{\ell },S\right)$, in (63) we use (36), the joint typicality lemma and I(V;XS)=0 and (65) follows from (60) and (61). Thus, (65) tends to zero exponentially fast for sufficiently small δ(ϵ).

Case (b): $R\geq \min\{I\left(X_{1};S\right),\ldots ,I\left(X_{\ell };S\right)\}$. We may assume without loss of generality that $R\geq I(X_{1};S)$. Now, we upper bound (43) by

(66) $$\begin{array}{c} e_{\vec{X_{\ell }}}\left(m,s\right)\leq \sum_{\hat{m}:\left(x\left(m\right),x\left(\hat{m}\right),s\right)\in T_{\epsilon }^{\left(n\right)}\left(X,X_{1},S\right)}P\left\{\;\left(x\left(m\right),x\left(\hat{m}\right),s,V\right)\;\in \;T_{\epsilon }^{\left(n\right)}\left(X,X_{1},S,V\right)\;\right\}. \end{array}$$

Note that by (35), we may narrow the distributions to those with

(67) $$I\left(X;X_{1}S\right)<R-I\left(X_{1};S\right)+\delta \left(\epsilon \right).$$

Therefore,

(68) $$\begin{array}{cc} e_{{\vec{X}}_{\ell }}\left(m,s\right) & \leq \exp\{n\left[|R\;-\;I\left(X_{1};XS\right){|}^{+}\;\;-I\left(V;X_{1}|XS\right)\;+\;2\delta \left(\epsilon \right)\right] \end{array}$$

(69) $$\begin{array}{c} \leq \;\exp\left(n\left[R\;-\;I\left(X_{1};XS\right)\;-\;I\left(V;X_{1}|XS\right)\;+\;2\delta \left(\epsilon \right)\right]\right) \end{array}$$

(70) $$\begin{array}{c} =\;\exp(n\left[R-I\left(X_{1};XSV\right)+2\delta \left(\epsilon \right)\right]) \end{array}$$

where (68) follows from (34) and the joint typicality lemma, (69) follows since by $R\geq I(X_{1};S)$ and (67) we have

(71) $$\begin{array}{cc} R & >I\left(X;X_{1}S\right)+I\left(X_{1};S\right)-\delta \left(\epsilon \right) \end{array}$$

(72) $$\begin{array}{c} =I\left(X;X_{1}|S\right)+I\left(X_{1};S\right)-\delta \left(\epsilon \right) \end{array}$$

(73) $$\begin{array}{c} =I\left(X_{1};XS\right)-\delta \left(\epsilon \right). \end{array}$$

Let $Z=X+S+V-X_{1}$. From (46)–(47), we get

(74) $$\begin{array}{cc} I(X_{1};XSV) & \geq I(X_{1};X_{1}+Z) \end{array}$$

(75) $$\begin{array}{c} =C\left(\frac{1}{EZ^{2}}\right) \end{array}$$

(76) $$\begin{array}{c} \geq C\left(\frac{1}{\Lambda +\sigma^{2}}\right) \end{array}$$

Using this result in (70), we obtain

(77) $$e_{{\vec{X}}_{\ell }}\left(m,s\right)\leq \exp\left\{n\left[R-C\left(\frac{1}{\Lambda +\sigma^{2}}\right)+2\delta \left(\epsilon \right)\right]\right\}$$

meaning that $e_{{\vec{X}}_{\ell }}\left(m,s\right)$ is exponentially vanishing if δ(ϵ) is sufficiently small and the rate condition in (30) holds.

Now, we consider error probability $P_{L}$. Define ${\vec{X}}_{L}=\left(XX_{1}X_{2}\ldots X_{L}SV\right)$. Let $V_{L}$ denote a finite ϵ-dense subset of Gaussian vectors ${\vec{X}}_{L}$ with variances at most $1,1,1,\ldots ,1,\Lambda ,\sigma^{2}$. Thus, $P_{L}$ can be upper bounded by

(78) $$\sum_{{\vec{X}}_{L}\in V_{L}}\frac{1}{N}\sum_{m=1}^{N}e_{{\vec{X}}_{L}}\left(m,s\right)$$

where

(79) $$\begin{array}{c} e_{{\vec{X}}_{L}}\left(m,s\right)=P\{\left(x\left(m\right),x\left(m_{1}\right),\ldots ,x\left(m_{L}\right),s,V\right)\;\in \;T_{\epsilon }^{\left(n\right)}\left({\vec{X}}_{L}\right),\\ \;\mathrm{for}\;\mathrm{some}\;\left(m_{1},\ldots ,m_{L}\right)\in S_{L}\;\mathrm{and}\;m_{\ell }\;\neq \;m\;\mathrm{for}\;\mathrm{all}\;\ell \in \left[L\right]\}. \end{array}$$

Thus, we need to show that $\frac{1}{N}\sum_{m=1}^{N}e_{{\vec{X}}_{L}}\left(m,s\right)$ vanishes as n→∞ for all Gaussian vectors ${\vec{X}}_{L}$ that satisfy (44)–(47) for all ℓ∈[L] and

(80) $$\begin{array}{c} EX_{L}^{2}=1,\;\left(X_{1},\ldots ,X_{L}\right)\;\mathrm{are}\;\mathrm{independent}, \end{array}$$

where (80) follows from (31) in which $\mathrm{Cov}(X_{i}X_{j})=0$ for all i≠j∈[L].

Observe that if $I(XV;SX_{1}\ldots X_{L})=0$, then we would have for all ℓ∈[L]

(81) $$\begin{array}{cc} 0 & =EX_{\ell }\left(X+S+V-X_{\ell }\right) \end{array}$$

(82) $$\begin{array}{c} =EX_{\ell }\left(S-X_{\ell }\right) \end{array}$$

(83) $$\begin{array}{c} =EX_{\ell }S-1, \end{array}$$

where (83) follows from (31).

Since $EX_{i}X_{j}=0$ for all i,j∈[L] and i≠j, the covariance matrix of $\left(S,X_{1},\ldots ,X_{L}\right)$ is equal to

(84) $$\begin{array}{c} \left[\begin{array}{cc} ES^{2} & 1_{L}^{T}\\ 1_{L} & I_{L} \end{array}\right] \end{array}$$

which has the determinant of $ES^{2}-L$. This determinant should be positive since the covariance matrix $\mathrm{Cov}(S,X_{1},\ldots ,X_{L})$ is positive definite. However, since $ES^{2}\leq \Lambda$, this assumption contradicts the assumption that Λ<L in (29). Thus, there exists η>0 such that

(85) $$\eta \leq I\left(XV;SX_{1}\ldots X_{L}\right)=I\left(XV;X_{1}\ldots X_{L}|S\right)$$

where we have used the fact that I(XV;S)=0.

Now, we may consider two cases $R<\min\{I\left(X_{1};S\right),\ldots ,I\left(X_{L};S\right)\}$ and $R\geq \min\{I\left(X_{1};S\right),\ldots ,I\left(X_{L};S\right)\}$. Therefore, using an identical argument as in the cases (a) and (b) for $P_{\ell }$, it follows that $e_{{\vec{X}}_{L}}$ is also exponentially vanishing.

## Figures and Tables

**Table 1:** References’ Summary.

Reference	Discrete/Gaussian	Shared Randomness	Cost Constraints	Max/Avg	List Decoding	Adversarial Model	Capacity or Notes
[1]	Discrete	✓	✗	Avg	✗	Oblivious	Blackwell et al. introduced AVC.
[3]	Discrete	✓	✗	Max and Avg	✗	Oblivious	Capacity remains the same for Max. error Probability.
[4]	Discrete	✗	✗	Max and Avg	✗	Oblivious	Studied deterministic capacity in a dichotomy theorem.
[5]	Discrete	✓	✗	Avg	✗	Oblivious	Found the necessary condition for the positive deterministic capacity by defining symmetrizability.
[6]	Discrete	✗	✓	Avg	✗	Oblivious	$\left\{\begin{array}{cccc} \max_{\begin{array}{c} P:g(P)\leq \Gamma \\ \Lambda_{0}\left(P\right)\geq \Lambda \end{array}} & \min_{\begin{array}{c} Y:P_{YXS}\in C_{0}\;\mathrm{for}\\ \mathrm{some}\;S,\;\mathrm{with}\;P(X)=P \end{array}} & I\left(X;Y\right), & \max_{P:g(P)\leq \Gamma }\Lambda_{0}\left(P\right)>\Lambda \\ 0, & & & \max_{P:g(P)\leq \Gamma }\Lambda_{0}\left(P\right)<\Lambda . \end{array}\right.$
[6]	Discrete	✗	✗	Avg	✗	Oblivious	$\max_{P}\;\min_{\begin{array}{c} Y:P_{YXS}\in C_{0}\;\mathrm{for}\\ \mathrm{some}\;S,\;\mathrm{with}\;P(X)=P \end{array}}I\left(X;Y\right)$ if and only if C>0
[7]	Discrete	✓	✓	Avg	✗	Oblivious	$\max_{X:Eg(X)\leq \Gamma }\;\min_{S:ES\leq \Lambda }I\left(X;Y_{X,S}\right)$
[8]	Discrete	✗	✓	Avg	✗	Oblivious	Capacity with general alphabets and states.
[9]	Discrete	✓	✗	Max	✗	Myopic	$\max_{P(x)}\;\min_{V\in W}I\left(P,V\right)$ *W* is a set of transition matrices from *X* to *Y*
[10]	Discrete	✓	✓	Max	✗	Delay	$\max_{P(x)}\;\min_{Q\in Q}I\left(P,W_{Q}\right)$ Q is a set of transition matrices from *X* to *Y*
[11]	Discrete	✗	✓	Max and Avg	✗	Causal	Upper bounds on the capacity
[19]	Discrete	✗	✗	Avg	✓	Oblivious	$\left\{\begin{array}{cccc} \max_{P} & \min_{\begin{array}{c} P_{YXS}:P_{YXS}\in C_{0}\;\mathrm{for}\\ \mathrm{some}\;S,\;\mathrm{with}\;P(X)=P \end{array}} & I\left(X;Y\right), & L>M\\ 0, & & & L\leq M. \end{array}\right.$
[20]	Discrete	✓	✓	Max	✓	Oblivious	$\max_{P\in P(X)}\;\min_{U\in U(P,\Lambda )}I\left(P,\sum W\left(y,x,s\right)U\left(s|x\right)\right)$
[20]	Discrete	✗	✓	Avg	✓	Oblivious	Upper and lower bounds on the capacity
[21]	Gaussian	✓	✓	Max	✗	Oblivious	$C\left(\frac{P}{\Lambda +\sigma^{2}}\right)$
[22]	Gaussian	✗	✓	Avg	✗	Oblivious	$\left\{\begin{array}{cc} C\left(\frac{P}{\Lambda +\sigma^{2}}\right), & P>\Lambda \\ 0, & P\leq \Lambda . \end{array}\right.$
This Paper	Gaussian	✗	✓	Avg	✓	Oblivious	$\left\{\begin{array}{cc} C\left(\frac{P}{\Lambda +\sigma^{2}}\right), & L>\frac{\Lambda }{P}\\ 0, & L<\frac{\Lambda }{P}. \end{array}\right.$

## Appendix A. Proof of Lemma 1

In order to prove (33), we use our proof in Reference [31] (Lemma 6) for one codebook. Moreover, to obtain (34)–(37), we apply the corresponding proof of these four equations in Reference [19] (Lemma 1) for Gaussian distributions. Note that Reference [19] focuses on discrete alphabets but the same proofs can be extended to Gaussian distributions by quantization of the set of continuous random variables in the following way.

Let $X_{i}$ be Gaussian i.i.d. *n*-length random vector (codebook) independent from each other with Var(X)=1. Fix $x\in T_{\epsilon }^{\left(n\right)}\left(X\right),s\in S^{n}$ and a covariance matrix $\mathrm{Cov}\left(X,X_{1},\ldots ,X_{\ell },S\right)\in V^{\left(\ell +2)\times (\ell +2\right)}$ such that $S^{n}$ is a ν-dense subset of $R^{n}$ for s such that ${\left||s|\right|}^{2}\leq n\Lambda$ and $V^{\left(\ell +2)\times (\ell +2\right)}$ is a ν-dense subset of $R^{\left(\ell +2)\times (\ell +2\right)}$ for positive definite covariance matrices with diagonals at most (1,1,…,1,Λ).

Using the similar proof in Reference [19] (Lemma 1), we obtain for given x,s and covariance matrix $\mathrm{Cov}(X,X_{1},\ldots ,X_{\ell },S)$ that the complement of each event in (34)–(37) happens with decreasingly doubly exponential probability for sufficiently large *n* meaning that

(A1) $$\begin{array}{c} P\left\{|\left\{m_{1}:\left(x,x\left(m_{1}\right),s\right)\in T_{\epsilon }^{\left(n\right)}\left(X,X_{1},S\right)\right\}|\leq \exp\left\{n\left[|R-I\left(X_{1};XS\right){|}^{+}+\delta \left(\epsilon \right)\right]\right\}\right\}\\ \;<\exp(-\exp(n\sigma (\epsilon ))), \end{array}$$

(A2) $$\begin{array}{c} P\left\{\frac{1}{N}|\left\{\;m\;:\;\left(x\left(m\right),x\left(m_{1}\right),s\right)\;\in \;T_{\epsilon }^{\left(n\right)}\;\left(X,X_{1}\;,S\right)\;\mathrm{for}\;\mathrm{some}\;m_{1}\;\neq \;m\;\right\}|\leq \;2\exp\left\{\;-n\delta \left(\epsilon \right)/2\;\right\}\right\}\\ \;<\exp\left(-\exp\left(n\sigma \left(\epsilon \right)\right)\right),\;\mathrm{if}\;I\left(X;X_{1}S\right)\;\geq \;{\left|R\;-\;I\left(X_{1};S\right)\right|}^{+}\;\;+\;\delta \left(\epsilon \right), \end{array}$$

(A3) $$\begin{array}{c} P\left\{\left|\left\{\;\left(m_{1},\ldots ,m_{\ell }\right)\;:\;\left(x,x\left(m_{1}\right),\ldots ,x\left(m_{\ell }\right),s\right)\in T_{\epsilon }^{\left(n\right)}\left(X,X_{1},\ldots ,X_{\ell },S\right)\right\}\right|\leq \exp\left\{n\delta (\epsilon )\right\}\right\}\\ \;<\exp\left(-\exp\left(n\sigma \left(\epsilon \right)\right)\right)\;\mathrm{if}\;R<\min_{k\in \{1,\ldots ,\ell \}}I\left(X_{k};S\right), \end{array}$$

(A4) $$\begin{array}{c} P\{\frac{1}{N}|\left\{m:\left(x\left(m\right),x\left(m_{1}\right),\ldots ,x\left(m_{\ell }\right),s\right)\in T_{\epsilon }^{\left(n\right)}\left(X,X_{1},\ldots ,X_{\ell },S\right)\;\mathrm{for}\;\mathrm{some}\;m_{1},\ldots ,m_{\ell }\neq m\right\}|\\ \leq \exp\left\{-n\delta \left(\epsilon \right)/2\right\}\}<\exp\left(-\exp\left(n\sigma \left(\epsilon \right)\right)\right)\mathrm{if}\;I\left(X;X_{1}\ldots X_{\ell }S\right)\;\geq \;\delta \left(\epsilon \right)\;\mathrm{and}\;R\;<\;\min_{k\in \{1,\ldots ,\ell \}}I\left(X_{k};S\right). \end{array}$$

Then, in order to complete the proof, since for any fixed ν the cardinality of finite set $S^{n}$ is only increasingly exponentially in *n* and the set $V^{\left(\ell +2)\times (\ell +2\right)}$ is finite along with the doubly decreasing exponential probabilities in (A1)–(A4), we derive that with probability approaching to 1, all inequalities in (34)–(37) hold simultaneously for sufficiently large *n*. Since these inequalities hold for every element in the finite sets $S^{n}$ and $V^{\left(\ell +2)\times (\ell +2\right)}$, then for any vector s,x and any given covariance matrix $\mathrm{Cov}(X,X_{1},\ldots ,X_{\ell },S)$ (with ${∥x∥}^{2}=n,{∥s∥}^{2}\leq n\Lambda$) which is not in its corresponding ν-dense subset, there exists a point in the corresponding ν-dense subset that is close enough to it (in its ν distance neighborhood). Now, by using the continuity properties of all sets, we may conclude that (34)–(37) hold also for any point which is not in the ν-dense subset.
